# CD74-AKT Axis Is a Potential Therapeutic Target in Triple-Negative Breast Cancer

**DOI:** 10.3390/biology13070481

**Published:** 2024-06-28

**Authors:** Jingchao Wang, Daoyuan Huang, Thu Anh Thai Nguyen, Liem Minh Phan, Wenyi Wei, Abdol-Hossein Rezaeian

**Affiliations:** 1 Department of Pathology, Beth Israel Deaconess Medical Center, Harvard Medical School, Boston, MA 02215, USA; 2 Genome and Systems Biology Degree Program, College of Life Science, National Taiwan University, Taipei 10617, Taiwan; 3 David Grant USAF Medical Center, Clinical Investigation Facility, 60th Medical Group, Travis Air Force Base, Fairfield, CA 94535, USA

**Keywords:** FAS (CD95), CD74, AKT, triple-negative breast cancer (TNBC), chemoresistance, apoptosis, peptidomimetic drug

## Abstract

**Simple Summary:**

Triple-negative breast cancer is a highly aggressive type of invasive breast cancer in which the patients frequently develop resistance to inhibitors used for induction of cell death. Therefore, restoring the cell death ability would improve the elimination of triple-negative breast cancer cells. Our preliminary data shows that the molecule “invariant chain” is highly expressed and inhibits death receptor signaling in triple-negative breast cancer. Interestingly, this survival pathway is regulated by “protein kinase B”, which has been aberrantly activated in 70% of breast cancers. Mechanistically, the activation of “protein kinase B” is likely critical for maintaining the protein stability and expression of the “invariant chain” to favor its oncogenic functions such as regulating cancer stem cells and epithelial–mesenchymal transition. We also found that selective sensitization of triple-negative breast cancer cells to death receptors using “invariant chain”-derived peptides could enhance death signaling. Moreover, the combination of the peptides with inhibitors for inactivation of “protein kinase B” could synergistically kill triple-negative breast cancer cells. These findings are innovative and impactful because they address important questions that not only reveal novel insight into how complex cell-death signaling is regulated but also provide detailed therapeutic approaches for the treatment of triple-negative breast cancer patients.

**Abstract:**

Triple-negative breast cancer (TNBC) cells are often resistant to FAS (CD95)-mediated apoptosis, but the underlying molecular mechanism(s) is not fully understood yet. Notably, the expression of the type II transmembrane protein, CD74, is correlated with chemotherapy-resistant and more invasive forms of cancers via unknown mechanisms. Here, we analyzed gene expression pattern of cancer patients and/or patient-derived xenograft (PDX) models and found that mRNA and protein levels of CD74 are highly expressed in TNBC and correlated with cancer stem cells (CSCs) and epithelial–mesenchymal transition (EMT) properties. Mechanistically, we found that AKT activation is likely critical for maintaining CD74 expression and protein stability to favor its oncogenic functions. Physiologically, epidermal growth factor (EGF) along with CD74 could activate AKT signaling, likely through binding of phosphorylated AKT (S473) to CD74, whereas inhibition of AKT could impair stability of CD74. We also revealed that CD74 binds to FAS and interferes with the intrinsic signaling of FAS-mediated apoptosis. As such, selective targeting of the CD74/FAS complex using the AKT inhibitor along with the CD74-derived peptide could synergistically restore and activate FAS-mediated apoptosis. Therefore, our approach of mobilizing apoptosis pathways likely provides a rationale for TNBC treatment by targeting the CD74/FAS and CD74-AKT axes.

## 1. Introduction

Triple-negative breast cancer (TNBC) is the most aggressive breast cancer subtype, responsible for 30% of all breast cancer deaths [1]. Although TNBC tumors are relatively sensitive to chemotherapy initially, patients develop resistance [2]. Moreover, it is thought that the cancer stem cell (CSC) population in tumor cells is potentially responsible for chemoresistance, clinical dormancy, and metastatic recurrence after surgery and adjuvant treatment [3]. In fact, deregulation of distinct cell-intrinsic processes, such as apoptosis [4], growth factor signaling [5], DNA repair [6], and alterations in the levels of drug transporter proteins [7] have previously been reported to be associated with chemoresistance. Moreover, it is reported that TNBC cells are often resistant to FAS-mediated apoptosis [8,9], but the underlying molecular mechanism is not fully understood [10]. In type I apoptosis signaling, the binding of FAS-L to FAS induces the formation of the death-inducting signaling complex (DISC), which involves the binding of the FAS-associated protein with FAS-associated death domain (FADD) protein and subsequent activation of procaspase-8 [11,12,13,14,15]. In type-II apoptosis signaling, the activation of procaspase-8 initiates apoptosis signals via mitochondria with the release of cytochrome c for the activation of caspase-9 [16,17,18]. Resistance to FAS-mediated apoptosis can be caused by low expression levels of FAS, overexpression of the cellular FLICE (FADD-like interleukin-1 beta-converting enzyme) inhibitory protein (cFLIP), and the X-linked inhibitor of apoptosis protein (XIAP) [19]. Furthermore, cancer cells infrequently express elevated levels of cFLIP or a mutant form of FAS/FAS-L, which can also cause resistance to FAS-mediated apoptosis [20]. Similarly, we previously found that PML/RARα binds to FAS and suppresses FAS-mediated apoptosis [21]. We also found that CD74 interferes with the expression of FAS receptors via a yet unknown mechanism [22]. Therefore, we anticipate that selective disruption of the CD74/FAS complex and restoring FAS-mediated apoptosis would improve the elimination of chemoresistant CSCs and TNBC cells [23].

The type II transmembrane protein, CD74, is expressed on antigen-presenting cells and was initially demonstrated to function as a MHC class II chaperone [24]. At steady state, CD74 is expressed on many cell types and serves as a receptor for proinflammatory cytokines, and the macrophage migration inhibitory factor (MIF) [25,26]. In macrophages, MIF binding to the extracellular domain of CD74 induces a downstream signaling cascade that results in a plethora of cellular events including activation of mitogen-activated protein kinase (MAPK) pathway, regulation of prostaglandin production, nuclear transcription initiation, cell proliferation, and cell survival [27,28]. Given its critical cellular functions, CD74 expression also correlates with more invasive forms of cancers and was found to be a highly and differentially expressed protein in chemotherapy-resistant cells via unknown mechanisms [29,30]. To this end, it was shown that CD74-cytosolic fragment (CD74-ICD) is released and translocated to the nucleus, which is activated by intramembrane proteolytic cleavage [31,32], probably through the ubiquitin-proteasome system for degradation process [31,32]. Notably, CD74-ICD translocates to the nucleus, where it induces downstream NF-κβ transcriptional activation for cell proliferation and survival [31,32,33,34,35]. This event in response to internal or environmental cues, might be crucial for awakening and proliferation of chemoresistant CSCs in the tumor microenvironment after chemotherapy. It is also reported that the PI3K/AKT oncogenic signaling pathway also plays a major role in breast cancer cell survival, metabolism, proliferation, and drug resistance [36,37,38]. Interestingly, the activation of PI3K/AKT, MIF and CD74 are associated with chemoresistance, tumor progression, and metastasis via unknown mechanisms [39,40]. Mechanistically, MIF induces AKT activation [28], and this effect is abolished by knocking down either *CD74* or *AKT* [41]. More interestingly MIF-stimulated CD74-dependent AKT activation is blocked by anti-CD74 antibodies [42], suggesting that activation of CD74 is likely associated with the activation of AKT, but the detailed mechanism remains largely elusive. Therefore, we anticipate that AKT-mediated intracellular activation of CD74 might be a critical oncogenic signal and thus blocking of CD74 and/or inactivation of AKT will enhance apoptosis in chemoresistant CSCs and TNBC cells.

Although we previously determined that CD74 exclusively associates with activation-resistant FAS through analysis of primary cell apoptosis in the patients with non-Hodgkin’s lymphoma [22], these new findings were observed in TNBC cells and are highly significant to target CD74/FAS and CD74/AKT axes with novel approaches to inhibit aggressive breast cancer cells and improve the outcome of patients. We also revealed a critical role of CD74 in the regulation of TNBC cells’ resistance to FAS-mediated apoptosis, which will provide, for the first time, a description of extracellular and intercellular mediators of TNBC cells’ resistance controlled by CD74 that is primarily sustained by inhibition of FAS-mediated apoptosis. Furthermore, we identified a high-affinity FAS binding peptide capable of disrupting the CD74-FAS complex and/or enhancing FAS-mediated apoptosis.

## 2. Materials and Methods

### 2.1. Antibodies

Anti-phospho-AKT1 (T308) antibody, anti-phospho-AKT1 (Ser473) antibody, anti-AKT, anti-phospho-S6 S235/236 (pS6), anti-pGSK3b (S21/9), anti-Myc, cleaved caspase 3 and epithelial–mesenchymal transition (EMT) antibody sampler kit were all from Cell Signaling Technology (CST) (Danvers, MA, USA). Anti-CD74 (LN-2), anti-FAS (B-10) antibodies, agonistic anti-CD74 antibody (C-16), FAS-L antibody (Kay-10), peroxidase-conjugated anti-mouse secondary antibody (A-4416), peroxidase-conjugated anti-rabbit secondary antibody (A-4914), ubiquitin and protein agarose A/G bead suspension were purchased from Santa Cruz Biotechnology, Inc. (Dallas, TX, USA). Human activating anti-FAS antibody (CH-11) was from Sigma-Aldrich, Inc. (St. Louis, MO, USA) and the IN1 antibody which detects the cytosolic domain of CD74 was purchased from Abcam (Waltham, MA, USA). Finally, antibodies for HA and Myc were from Covance and Pierce, respectively.

### 2.2. Cell Culture and Reagents

MDA-MB-231, MDA-MB-436, and HEK293 cells were cultured in the Dulbecco-modified eagle medium (DMEM) supplemented with 10% fetal bovine serum (FBS), 100 units of penicillin, and 100 µg/mL streptomycin. All cell lines were purchased from the American Type Culture Collection (ATCC) (Manassas, VA, USA) and confirmed to be mycoplasma-free before experiments. TRAF6 shRNA expression lentiviral plasmids and shRNA for luciferase were described previously [43]. The AKT inhibitor (AZD5363) and MG132 were purchased from Selleck Chemicals (Houston, TX, USA) and Cell Signaling Technology (Danvers, MA, USA), respectively. For drug treatment, MDA-MB-231 cancer cells were starved and incubated with or without MIF (100 ng/mL) for 24 h and/or EGF (50 ng/mL) for 30–60 min. MIF signaling was blocked in cells using the ISO-1 antagonist (20 µM, Calbiochem (San Diego, CA, USA)) for the comparison study. The mutants of pEF4-CD74-myc were gifted from Idit Shachar (Weizmann Institute of Sciences) and HA-tagged *FAS* mutants were obtained from Addgene (Watertown, MA, USA). Patient-derived xenografts (PDXs) were provided by Jenny C Chang (Houston Methodist Research Institute). Overall, MDA-MB-231 cancer cells were treated with the agonistic anti-CD74 antibody (5 µg/mL, C-16, Santa Cruz (Dallas, TX, USA)) along with the isotype control or FAS-L (50 ng/mL, Enzo (New York, NY, USA)) for 1 h or with the AKT inhibitor (in the range of 0–8 µM, AZD5363) for 24 h. Moreover, breast cancer cells were incubated in 1 mL of serum-free medium including 5, 10, 25, 50, and 100 μM peptides for 24 h along with or without FAS-L (20 ng/mL) for 1 h. Random-sequence peptides of the same length and isoelectric point were used as controls. On the other hand, cancer cells were treated with humanized anti-CD74 antibody (hLL1, 5 µg/mL, Creative Biolabs (Shirley, NY, USA)) crosslinked with goat anti-mouse (GAM) or anti-human (GAH) IgG followed by 50 ng/mL of either agonistic anti-FAS antibody CH-11 or FAS-L (recombinant) for 24 h.

### 2.3. Lentiviral Packaging and Infection

Lentiviral shRNAs of TRAF6 and luciferase (control) were used, and each shRNA was co-transfected with packing (1VPR8.9) and envelope (VSV-G) plasmids into 293T cells using Lipofectamine 2000 reagent according to the manufacturer’s standard procedures. Virus particles containing TRAF6 or luciferase shRNA(s) were used to silence these genes in MDA-MB-231 cells. All infected cells were selected in the medium with appropriate antibiotics for 4 days to be stable according to a previously published protocol [44].

### 2.4. Immunoblotting (IB) and Immunoprecipitation (IP)

We activated MDA-MB-231 cells with the agonistic anti-FAS antibody (CH-11, 50 ng/mL for 24 h) and immunodepleted active FAS in the cell extracts using the CH-11. The remaining cell extract was precipitated by activation-resistant anti-FAS antibody (B-10) for the immunoblotting. For peptide treatment, MDA-MB-231 cells were incubated in the serum-free medium including 5, 10, 25, 50, and 100 μM peptides for 24 h, and the formation of CD74-FAS complexes was monitored by IP/IB analysis using anti-CD74 (LN-2) and anti-FAS (B-10) antibodies. For immunoblotting, cells were lysed in NP-40 lysis buffer (50 mM Tris-HCl, pH 7.5, 120 mM NaCl, 0.5% NP-40) supplemented with a protease inhibitor cocktail (cOmplete, Roche, Basel, Switzerland). The protein concentrations of lysates were analyzed by the Bio-Rad Protein Assay Dye (Hercules, CA, USA) [45]. For immunoprecipitation (IP), cells were subjected to lysis by E1A lysis buffer (250 mM NaCl, 50 mM HEPES (pH 7.5), 0.1% NP-40, 5 mM EDTA, protease inhibitor cocktail (Roche)). Random-sequence peptides of the same length and isoelectric point were used as controls.

### 2.5. Immunohistochemistry (IHC)

Patient-derived xenografts (PDXs) were formalin-fixed and paraffin-embedded for providing tissue sections according to the standard instructions [46]. For an in vivo study, C57BL/6 mice were transfected with 100 µg of CD74 or empty vector plasmid through tail vein injection [43]. Twenty-four hours later, the mice received a lethal dose of mouse anti-FAS antibody (Jo2, 0.4 μg/g; BD Biosciences (Woburn, MA, USA)) through intraperitoneal injection. The surviving mice were killed at the time of death to harvest liver tissues for formalin-fixed, paraffin-embedded liver tissue sections. To confirm that CD74 blocks FAS-mediated apoptosis, the liver sections were subjected to H&E, immunohistochemical, and TUNEL staining for observation by confocal microscopy (Carl Zeiss NTS GmbH, Cambridge, UK).

### 2.6. Cell Survival and FACS Assays

MDA-MB-231 cells were treated with 50 ng/mL CH-11 or 20 ng/mL FAS-L for 24 h followed by incubation with or without peptides (100 µM) for 24 h. Cells were then stained with Annexin V and FITC and the degree of early cell apoptosis was evaluated (in quadruplicate) using Annexin V-FITC apoptosis detection kit (BD Biosciences, Woburn, MA, USA)) according to the manufacturer’s instructions. The nonspecific binding of peptides was blocked by preincubating cells with off-target peptides. On the other hand, cell samples were stained with propidium iodide (PI) to be analyzed using a fluorescence-activated cell sorting FACSCalibur flow cytometer (BD Biosciences, Woburn, MA, USA) [43]. Data were analyzed and plotted by using Flow Jo version 7.5.5 software.

### 2.7. Bioinformatics and Statistical Analyses

The amplification and expression of *CD74* were assessed using data sets from The Cancer Genome Atlas (TCGA) (http://www.cBioPortal.org) and GENT (http://medicalgenome.kribb.re.kr) (Both were accessed on 1 February 2021). The breast cancer patient data set GSE10797 was downloaded from the Gene Expression Omnibus database. The cohort was analyzed with Nexus Expression, version number of 3.0 (BioDiscovery, Inc., Boston, MA, USA) and a Circos-map building tool, version number of 0.69-9 (Circos software, www.circos.ca (accessed on 1 February 2021)). The results are represented as mean ± standard deviation (SD). All statistical analyses are performed in GraphPad Prism Software (version 6.0c) using two-sided Student’s *t*-test for comparisons between two groups, and one-way ANOVA followed by Dunnett multiple comparison test for comparing combination treatment group with single agent-treated groups. *p* < 0.05 was considered a statistically significant difference.

## 3. Results

### 3.1. CD74 Expression Upregulates Cancer Hallmarks in Breast Cancer

**Figure 1:**
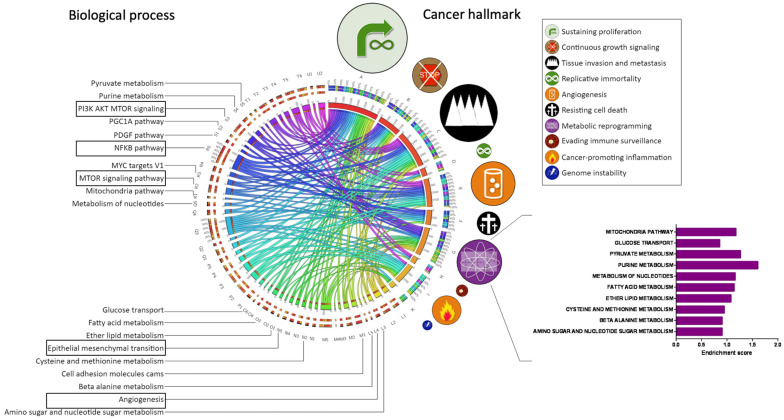
Overexpression of *CD74* is associated with upregulation of major cancer hallmarks. Transcriptomics gene expression profiles of breast cancer patients (cohort GSE10797, Gene Expression Omnibus database, http://www.ncbi.nlm.nih.gov/geo/) were accessed on 1 February 2021 and analyzed using the Nexus Expression 3.0 software (BioDiscovery). The gene expression profiles of the highest *CD74* quartile were compared with the lowest *CD74* quartile and matched with correlated biological processes and corresponding cancer hallmarks. The size of cancer hallmark symbols signifies the magnitude of their upregulation when *CD74* expression is elevated in breast cancer. The activated signaling pathways related to the PI3K/AKT/mTOR, NF-κβ, sustaining proliferation, epithelial–mesenchymal transition, tissue invasion, metastasis and angiogenesis are shown in the boxes. The enrichment score and the bars in the histogram represent the activated molecular metabolism upon the activation of CD74. This Circos map was built using the Circos software, version number of 0.69-9 (www.circos.ca (accessed on 1 February 2021)).

It was found that CD74 is expressed in lymphoma, multiple myeloma, leukemia, and thymic cancers [47,48,49,50,51,52]. It has also been previously shown that MDA-MB-231 cells are enriched with the population of CSCs [53]. Therefore, we strived to understand the possible physiological role of CD74 in solid tumors including breast cancer cells. To this end, we analyzed transcriptomics gene expression profiles of breast cancer patients in the Gene Expression Omnibus database and found that CD74 is upregulated and correlated with aberrant elevation of various indicated cancer hallmarks (Figure 1). We also found that CD74 is involved in several biological processes, including glucose uptake, metabolic reprogramming through activation of several signaling pathways such as PI3K/AKT/mTOR and NF-κβ signaling. In fact, CD74 upregulates the genes that are most responsible for the regulation of growth, survival, metabolism, proliferation, angiogenesis, epithelial–mesenchymal transition, and metastasis (Figure 1). For example, responsible genes in the regulation of cancer cell survival and proliferation such as *STAT1*, *STAT5A*, *CD44* and *BCL2* are upregulated (Supplementary Figure S1A). In contrast, genes in the regulation of DNA damage signaling and cell cycle are downregulated upon high expression levels of CD74 in breast cancer (Supplementary Figure S1B).

### 3.2. AKT Activation Is Likely Required for the Stabilization of CD74 in TNBC

**Figure 2:**
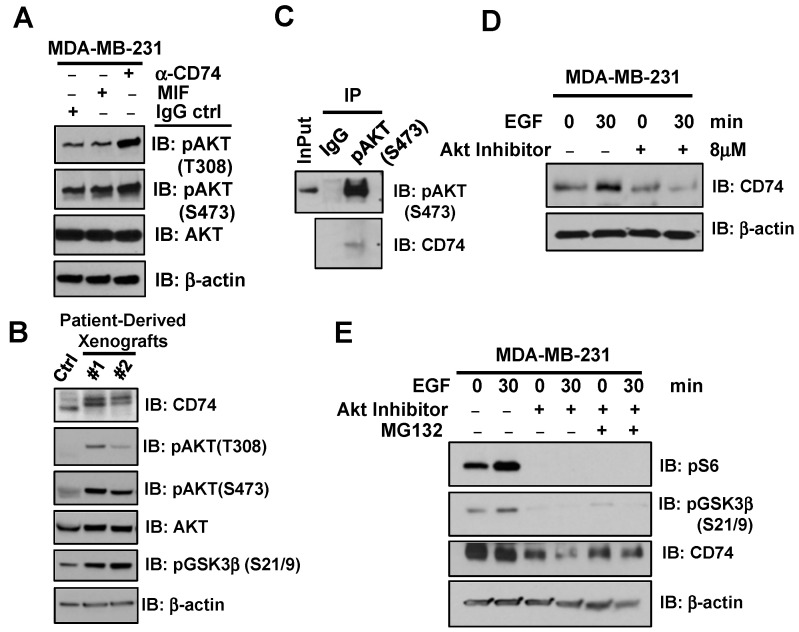
Inhibition of AKT signaling downregulates the protein abundance of CD74. (**A**) IB of AKT kinase activation in MDA-MB-231 cells pretreated w/wo MIF (100 ng/mL) along with the agonistic anti-CD74 antibody (5 µg/mL) or isotype control for 18 h. (**B**) IB analysis of activated AKT signaling correlated to CD74 expression in TNBC-driven PDX tumor compared to control (normal breast tissue). (**C**) Immunoprecipitation (IP) shows the binding of AKT-phosphorylation with CD74 in HEK-293 cells. (**D**) MDA-MB-231 cells incubated w/wo AKT inhibitor for 24 h, were treated by EGF (25 ng/mL), lysed, and subjected to IB. (**E**) Stabilization of CD74 is shown in MDA-MB-231 cells treated as panel D, w/wo MG132 for 5 h. Representative Western blot of two independent experiments.

It has been previously shown that MIF mediates CD74 induction for the regulation of the PI3K/AKT signaling [28,33], but the mechanism is not known in TNBC and breast cancer cells. We observed that CD74 activated AKT phosphorylation in MDA-MB-231 and MDA-MB-436 cells upon treatment with agonistic CD74 antibody (Figure 2A, Supplementary Figure S2A), suggesting that CD74 activates AKT signaling in this experimental setting. In support of this finding, immunoblotting of tumor lysates from TNBC-related patient-derived xenograft (PDXs), showed that in TNBC setting, elevated CD74 expression positively correlates with the activation of AKT signaling pathway (Figure 2B). In order to reveal a possible direct interaction of CD74 with AKT, we immunoprecipitated the lysates of HEK-293 cells with anti-phospho-AKT1 (Ser473) antibody, followed by immunoblotting with the IN1 antibody, which detects the cytosolic domain of CD74. We found that mTORC2-mediated phosphorylation of AKT (at S473) led to its interaction with the ICD domain of CD74 (Figure 2C). Moreover, CD74 could be induced upon EGF treatment while disruption of AKT signaling using the AKT inhibitor could diminish the induction of CD74 by EGF as a novel physiological stimulator for CD74 signaling (Figure 2D). We next incubated MDA-MB-231 cells with the AKT inhibitor along with and without EGF and MG132 (proteasome inhibitor) for the analysis of CD74 protein stability. Notably, we found that inactivation of AKT decreased the stability of the CD74 protein, while such reduction was largely rescued by MG132 (Figure 2E). This suggests that AKT activation is critical for maintaining CD74 expression and stability. In a recent phosphoproteomic study, the RXRXXS/T motif of over 300 downstream substrates has been recognized to be phosphorylated by the AGC family kinases including AKT [54]. Interestingly, we found that the cytosolic domain of CD74 in humans includes the RXRXXS/T motif along with a lysine (K14) residue that might be phosphorylated by AKT for the subsequent E3 ligase ubiquitylation (Supplementary Figure S2B). Previously, it was shown that the E3 ligase TRAF6 regulates AKT ubiquitination and activation [55]. In searching for a new E3 ligase for CD74 ubiquitylation, we found that TRAF6 binds to and polyubiquitinates CD74 upon EGF treatment (Supplementary Figure S2C). These results support the model that TRAF6 might be the upstream E3 ligase responsible for polyubiquitination and stabilization of CD74. However, further in-depth studies are warranted to reveal the critical role of TRAF6 in regulating CD74 protein ubiquitination, degradation and/or cleavage pathway.

### 3.3. Activation of CD74 Is a Possible New Prognostic Biomarker in Triple-Negative Breast Cancer

**Figure 3:**
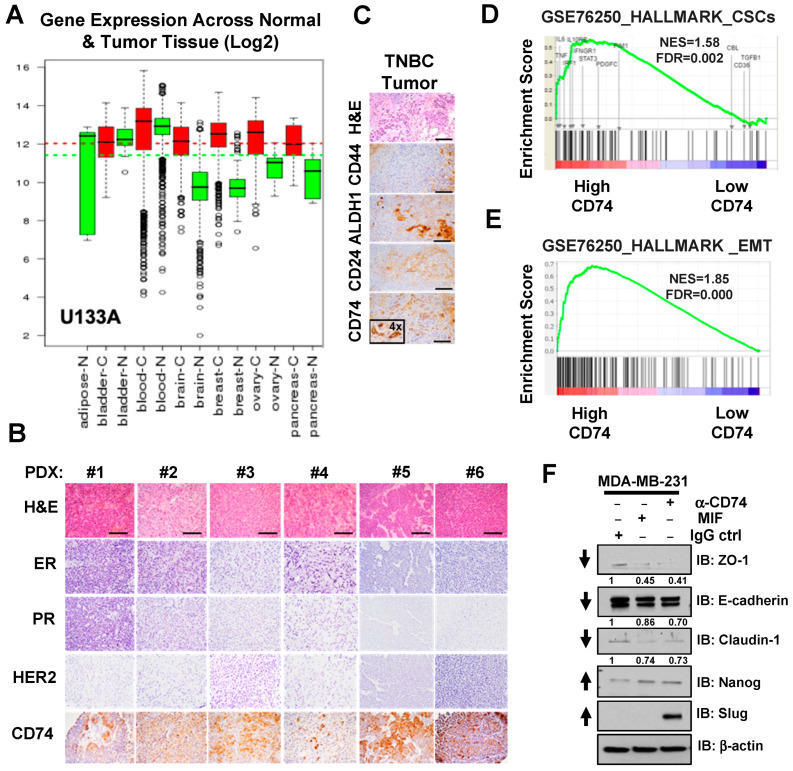
CD74 is overexpressed in TNBC that correlates with CSCs and EMT properties. (**A**) mRNA expression pattern of 34,000 samples was analyzed and profiled by Affymetrix U133A platforms. Box shows overexpression of CD74 in brain, breast, ovary, and pancreas cancer. N, normal (green) versus C, cancer (red). Green and red dashes represent the average expression of CD74 across normal and cancer tissues, respectively. (**B**,**C**) IHC analysis of CD74 (**B**) and CSCs markers. (**C**) in patient-derived Xenograft (PDX) tumors from TNBC patients (numbers 1–6). TNBC is characterized by the lack of ERα, PR, and HER2 expression, while CD74 is highly expressed. Scale bar = 100 µM. (**D**,**E**) Gene set enrichment analysis of cancer stem cells (CSCs) and epithelial-to-tesenchymal Transition (EMT) shows that these processes are significantly enriched in patients with high CD74 expression. (**F**) Lysates of MDA-MB-231 cells treated with or without MIF (100 ng/mL) and/or agonistic anti-CD74 antibody (5 µg/mL) or isotype control for 24 h, followed by IB for detection of EMT signaling. Arrows indicate downregulation and upregulation of EMT markers.

We found that CD74 is correlated with aberrant elevation of cancer hallmarks (Figure 1), but its biological impacts remain elusive. To specify whether CD74 is highly expressed in TNBC and regulates invasion, we first analyzed gene expression database of Normal and Tumor tissues in the National Genomics Data Center and found that CD74 is more differentially expressed in solid tumors of the breast, brain, blood, ovary, and pancreas when compared to corresponding non-malignant cells (Figure 3A). We next analyzed the expression of the CD74 in normal and tumor tissues using the TCGA database (PanCancer Atlas) and the UALCAN (The University of Alabama at Birmingham Cancer Data Analysis Portal) database, and we observed that copy number, mRNA and protein level of CD74 are upregulated in breast cancer and breast invasive carcinoma (Supplementary Figure S3). We further validated and performed immunohistochemical staining of TNBC tissues derived from 6 patient derived xenograft (PDX) models using CD74 antibody and found that CD74 is highly expressed in an aggressive form of breast cancer, TNBC (Figure 3B). Furthermore, the protein level of CD74 expression was found to be expressed along with ALDH compared to the moderate expression level of CD24 and CD44 in the PDX tumor models (Figure 3C). Of note, ALDH^+^ is a stronger indicator for migration and metastasis of CSCs, while the ratio of CD44/CD24 is critical for CSCs proliferation and tumorigenesis [53]. We next executed gene set enrichment analysis and found that expression of CD74 is significantly correlated with gene expression of CSCs and EMT markers in TNBC patients (Figure 3D,E). Next, we performed immunoblotting and further confirmed the correlation of CD74 with epithelial–mesenchymal Transition (EMT) through the downregulation of epithelial markers such as E-cadherin or upregulation of mesenchymal markers such as Slug and Nanog (Figure 3F) in the TNBC cell line, MDA-MB-321. These results suggest that overexpression of CD74 might be a new prognostic biomarker and critical for the regulation of CSCs and EMT for TNBC progression and metastasis. Notably, the high level of CD74 expression is observed in the blood (Figure 3A) that might pose challenges for its use as a prognostic biomarker, therefore the non-specificity of this marker and its limitations in clinical applications are concerning.

### 3.4. Synergistic Effect of CD74-Derived Peptide and AKT Inhibitor Sensitizes TNBC to FAS-Mediated Apoptosis

**Figure 4:**
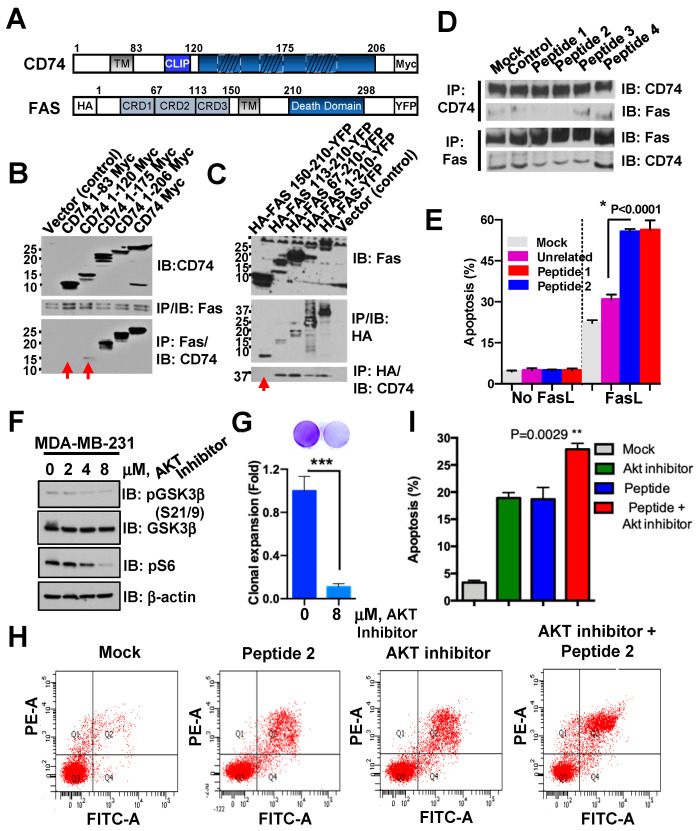
Targeting the inhibitory CD74-FAS complex using CD74-derived peptides synergize with AKT inhibitor to enhance early apoptosis in TNBC. (**A**) A schematic representation of the human CD74 and FAS structure with deletion mutants. TM; transmembrane, CLIP; class II-associated li chain peptide, and CRD; Cysteine Rich Domain. (**B**) Lysates of 293 cells transfected with Myc-CD74 mutants, were immunoprecipitated (IP) by the anti-FAS antibody, followed by immunoblotting (IB). The arrow shows defects in binding. (**C**) Lysates of 293 cells co-transfected with Full Length (FL)-CD74 and FAS mutants were IP with anti-HA antibody (FAS), followed by IB for detection of CD74. (**D**) IP followed by IB analysis of CD74-FAS complexes in MDA-MB-231 cells pre-treated with CD74-derived peptides for 24 h. The unrelated peptide was used as a negative control along with mock. (**E**) MDA-MB-231 cells post-treated with peptides for 24 h, were incubated with FAS-L (20 ng/mL) for 1 h and stained with propidium iodide (PI) for FACS analysis. (**F**,**G**) Lysates of MD-MB-231 cells treated with the indicated concentration of AKT inhibitor for 24 h were subjected to IB (**F**) and Coomassie blue staining (**G**). (**H**) MDA-MB-436 cells were incubated with CD74-derived peptide 2 along with AKT inhibitor (8 µM) or mock (control) for 24 h and stained with Annexin V-FITC for early apoptosis detection using flow cytometry and (**I**) the synergistic effect of each combination with peptide and/or AKT inhibitor was quantified. The quantified results are presented as mean ± S.D. of triplicate results using a two-tailed *t*-test. * *p* < 0.05, ** *p* < 0.01, *** *p* < 0.001 were considered significant.

We found that the amino acid sequence of CD74 is evolutionarily conserved among species (Supplementary Figure S4A). Moreover, CD74 is upregulated and correlated with cancer cell survival and growth (Figure 1). We previously reported that CD74 interferes with the expression of the FAS receptor on the surface of lymphoma cells [22]. However, whether CD74 directly binds with and regulates FAS-mediated apoptosis in TNBC has not been known yet. CD74 and FAS are integral membrane proteins with intrinsic and ectodomains, which are attached to the plasma membrane (Figure 4A). Herein, we investigated the location of the CD74-FAS-binding site and found that the CLIP-domain of CD74 binds to the cysteine-rich domain 3 (CRD3) of FAS in HEK-293 cells (Figure 4B,C). Furthermore, results from the mapping of the interacting domain of CD74 with FAS suggest that the FAS binding site is likely localized between residue 60 and 100 of CD74. We next explored the idea to develop peptide therapy for the naturalization of the interaction domain of CD74 and FAS to induce FAS-mediated apoptosis in TNBC. To this end, we designed 20 amino acid-long peptides that spanned the FAS-interaction region within CD74 (Figure 4A–C, Supplementary Figure S4B). We incubated cancer cells with these synthetic peptides (100 µM) for 24 h before harvesting cells for immune-precipitation and Western blot analysis. The results showed that the CD74-FAS complex could be disrupted with peptides 1 and 2 representing a putative binding domain of CD74 at the CLIP domain (Figure 4D). Interestingly, peptides from the binding domain of CD74 enhanced the sensitivity of cancer cells to FASL-induced apoptosis in MDA-MB-231 cells (Figure 4E). These results demonstrate that CD74 exclusively coprecipitated with activation-resistant FAS, suggesting a critical role for CD74 in preventing activation of FAS. Since we found that AKT activation is likely required for the stabilization of CD74 in TNBC (Figure 2), we incubated MDA-MB-231 cells with an AKT inhibitor (AZD5363, 1 µg/mL) for IB and proliferation assay. Notably, the phosphorylation of its substrates (e.g., GSK3β, S6) and cell proliferation were decreased upon AKT inhibition (Figure 4F,G). We next executed combination therapy to target CD74/FAS and CD74-AKT axes using the AKT inhibitor along with CD74-derived peptide. Interestingly, we found that CD74-derived peptide synergizes with AKT inhibitor to activate cleaved caspase 3 and enhance early apoptosis in MDA-MB-436 cancer cells (Figure 4H,I, Supplementary Figure S4C). Thus, CD74 is a potential inhibitor of FAS-mediated apoptosis and an attractive therapeutic target in TNBC cells.

### 3.5. CD74 Functions as an Oncogenic Signal to Block FAS-Mediated Apoptosis In Vivo

**Figure 5:**
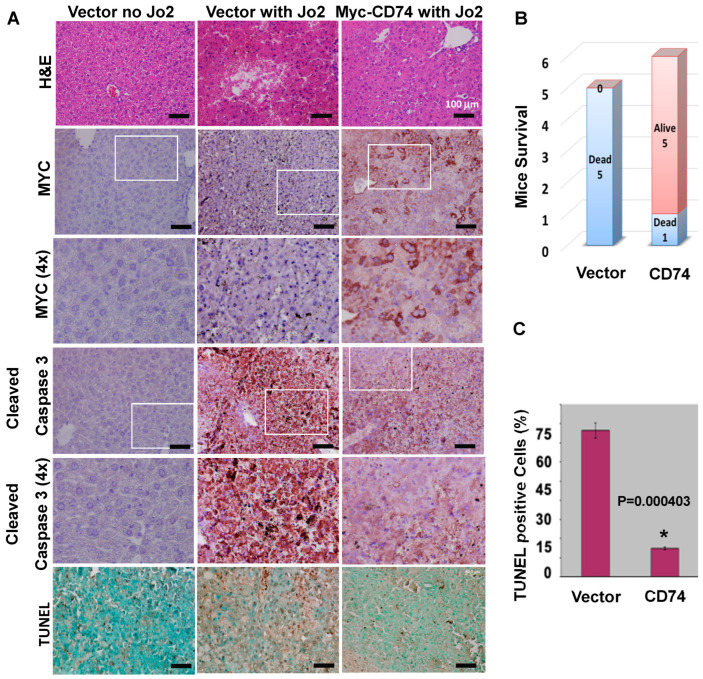
Oncogenic function of CD74 protects hepatocytes from a lethal dose of the agonistic FAS antibody (Jo2). (**A**) Immunohistochemistry of liver tissues from control (vector, *n* = 5) and Jo2-challenged (vector with Jo2 (*n* = 5) and CD74-Myc with Jo2, *n* = 6) mice. H&E; hematoxylin and eosin staining, Myc-tag staining (CD74). Hemorrhages and activation of Caspase 3 are shown to be inhibited with the expression of CD74 in the liver. (**B**) Survival number of challenged mice with Jo2 antibody at 6 h. Jo2, mouse agonistic anti-FAS antibody. (**C**) The percentage of apoptosis was measured from liver tissue using a TUNEL assay for positive staining cells. Scale bar = 100 µM. Each column represents the mean ± S.D. of three independent experiments, * *p* < 0.05 was considered significant.

We found that CD74 exclusively coprecipitated with activation-resistant FAS to indicate that CD74 has a critical function for prevention of FAS activation (Figure 4D,E). We next determined whether CD74 expression drives oncogenic signals through inhibition of FAS-mediated cell apoptosis in mice tissues. To this end, we intravenously injected mice with CD74-expressing and control plasmids to express transgenes in mouse liver. After induction of apoptosis with a lethal dose of mouse agonistic anti-FAS antibody (Jo2), we determined that CD74-transfected mice had relatively higher survival rates compared to vector-transfected mice with less active caspase 3 in the liver tissue (Figure 5A,B). In fact, we could reveal massive hemorrhages along with activation of caspase 3 in the hepatocytes after induction of FAS, while CD74 could inhibit FAS-mediated apoptosis in the liver determined by H&E, IHC and TUNEL staining (Figure 5A,C). Although further in-depth studies are needed, this finding indicates that CD74 likely functions as an oncogenic signal by possibly binding to FAS in mouse cells to confer resistance against FAS-mediated apoptosis in vivo.

## 4. Discussion

**Figure 6:**
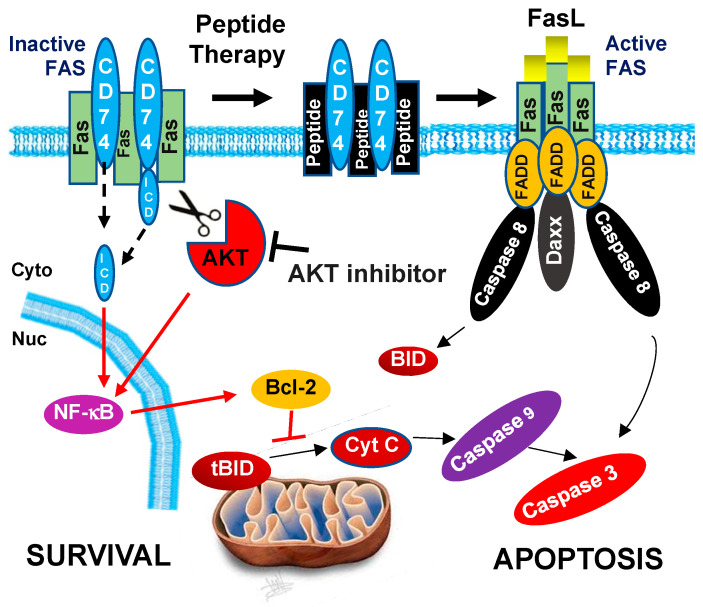
Proposed model of targeting CD74/FAS and CD74/AKT signaling for TNBC therapies. On one hand, the FAS/FAS ligand pathway has a central role in the regulation of programmed cell death or apoptosis through the binding of the FAS-associated protein with the FAS-associated death domain (FADD) for the subsequent activation of procaspase-8. On the other hand, CD74 mediates survival signals through the cleavage of CD74-cytosolic fragment (ICD), which transfers into the nucleus and activates NF-kB and results in high Bcl-2 levels for inhibition of mitochondrial apoptosis. Here we report that CD74 interferes with FAS-mediated apoptosis through direct binding of CD74 with FAS. Moreover, Inhibition of AKT signaling downregulates the protein stability of CD74. Therefore, we developed the idea that synergistic effect of using CD74-derived peptides and AKT inhibitors might sensitize TNBC to FAS-mediated apoptosis. Red arrows are oncogenic signals.

It is shown that anthracyclines (such as doxorubicin and epirubicin) induce or synergize with FAS to induce apoptosis [56,57,58]. However, high doses, which are required for the effective treatment of malignancies, induce cardiotoxicity with the cumulative dose in plasma [59,60,61]. More recently, ion chelator dexrazoxane has been approved for cardioprotection of women with metastatic breast cancer with a cumulative dose of DOX (above 300 mg/m^2^) [62], but the long-term cardioprotective effect of dexrazoxane has not been determined. On the other hand, the blockage of FAS-mediated apoptosis is considered a key mechanism of cell persistence [63,64,65]. Therefore, selective sensitization of the FAS inhibitor (blockage) to FAS-mediated apoptosis may increase the effectiveness of anthracyclines at doses below the cardiotoxic threshold. Here, we introduced CD74 as one of those crucial inhibitors for FAS-mediated apoptosis and our results point to the possibility that the FAS pathway is synergized and activated by the CD74-derived peptide and AKT inhibitor in breast cancer (Figure 4 and Figure 6). Possible mechanisms include blockage of CD74-mediated inactivation of FAS may release FAS from the inhibitory complex and facilitate FAS-mediated apoptosis.

Activation of the PI3K/AKT pathway is also well known in TNBC [66,67] and CSCs [68] associated with drug resistance through an unknown mechanism [39,40]. It was previously shown that agonistic anti-CD74 antibody and ligand (MIF) bind to the CD74-CD44 complex and activate AKT via an unknown mechanism [28,33]. Because CD74 is highly expressed in CSCs and TNBC cells (Figure 3), and this effect is abolished by silencing either CD74 or AKT [41,42], we hypothesized that inhibition of AKT might inactivate NF-κβ mediated Bcl-2 signaling downstream of CD74 which then results in activation of caspase 3 and 7 (type-II apoptosis) [16,17,18]. This complements the findings that disruption of the CD74/FAS complex using CD74-derived peptides might release FAS from the inhibitory complex for activation of caspase 8 (type-I apoptosis). Herein we discovered the role of the CD74/AKT axis in chemoresistant TNBC cells for the enhancement of apoptosis using an AKT inhibitor alone or in combination with CD74-derived peptides in vitro.

Therapeutic peptides are a promising approach to treat many diseases including cancer [69,70,71,72,73,74,75,76]. They are (a) easy to synthesize, (b) have a high target specificity, and selectivity, and (c) have low toxicity [77]. CD74-derived peptides are expected to associate with FAS and to prevent the formation of CD74-FAS complexes on the outer cell membrane as we have shown for 2 of 6 tested 20 residue peptides (Figure 4, Supplementary Figure S4B). However, 20 mer peptides can contain folding patterns that might cause nonspecific activity, or negatively affect the conformation of the FAS receptor, similar to full-length CD74; thus, shorter and more drug-like peptides will be essential for testing their ability to disrupt CD74-FAS complexes to sensitize TNBC cells to FAS-induced apoptosis. Of note, the therapeutic potential of peptides and peptidomimetic drugs has been recently demonstrated for numerous protein–protein interactions and other targets including p53-MDM2 and BH3-mimicking agents [78,79,80,81]. Herein, obtaining detailed structure-activity data and an optimized linear peptide will provide the basis for further studies applying cyclization strategies for iteratively converting peptides into more drug-like compounds [82,83].

## 5. Conclusions

Overall, we identified novel functions of CD74 in the deregulation of FAS-mediated apoptosis in MDA-MB-231 chosen as the cell line model for TNBC for developing novel inhibitors to selectively target oncogenic functions of CD74 based on mechanisms we have identified. We described signaling defects underlying apoptosis resistance and their reversal approach by targeting aberrant CD74 and/or AKT activation in TNBC in vitro. Our results will likely have a great impact through development of targeted therapies with unique peptides or humanized anti-CD74 antibody to eliminate the side effects of chemotherapy which is widely used for the treatment of TNBC patients with cardiotoxicity. The results also enable us to develop cell-penetrating-peptides (CPPs) to facilitate cellular intake of AKT inhibitors associated by cargo; and/or to establish unique CD74 antibody-drug conjugate (ADC) with AKT inhibitor to target CD74-positive cancer cells selectively and efficiently. However, additional in-depth mouse studies are required to reveal the synergistic effects of peptide therapy with the AKT inhibitor to induce FAS-mediated apoptosis in vivo as a novel TNBC therapy.

## Data Availability

All data are contained within the article.

## Supplementary Materials

The following supporting information can be downloaded at: https://www.mdpi.com/article/10.3390/biology13070481/s1, Figure S1: Venn diagram reveals the oncogenic impact of CD74 signaling; Figure S2: TRAF6 might function as an E3 ubiquitin ligase for ubiquitylation of CD74; Figure S3: CD74 is highly amplified and overexpressed in several types of human cancers; Figure S4: CD74 amino acids sequences are conserved among species.
