# Supplementary material for: CD74-AKT Axis Is a Potential Therapeutic Target in Triple-Negative Breast Cancer
Source: Biology (Basel). 2024 Jun 28;13(7):481. doi: 10.3390/biology13070481 (PMC11274071; doi:10.3390/biology13070481)
Supplement: Supplementary file 1 [file biology-13-00481-s001.zip › biology-3024455-supplementary.pdf]

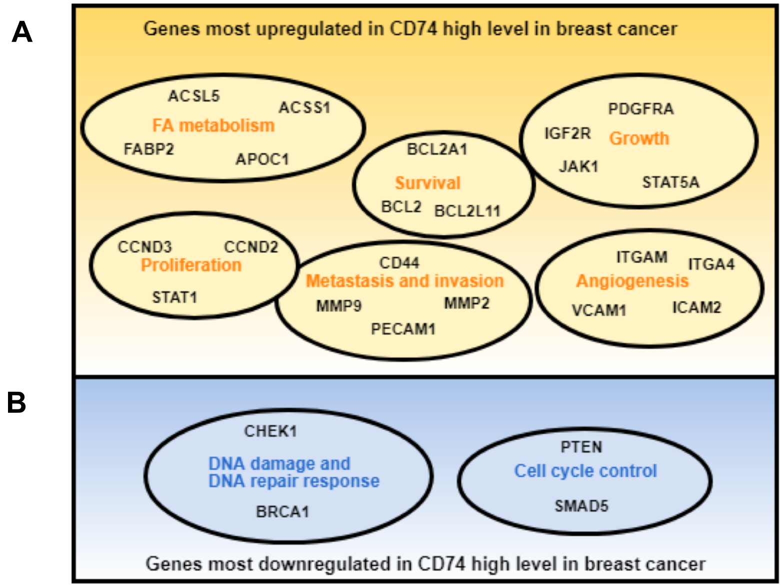


**Supplementary Figure S1**

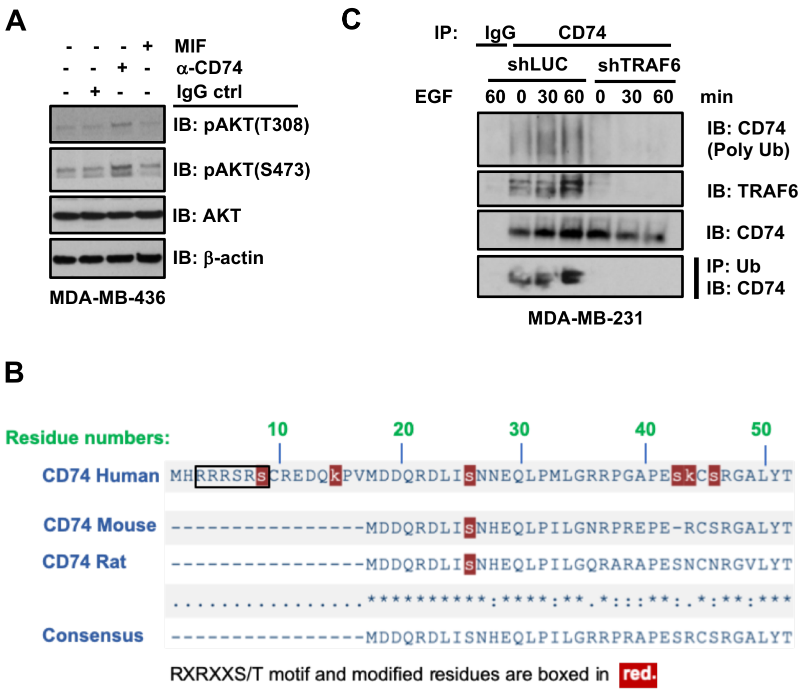


**Supplementary Figure S2**

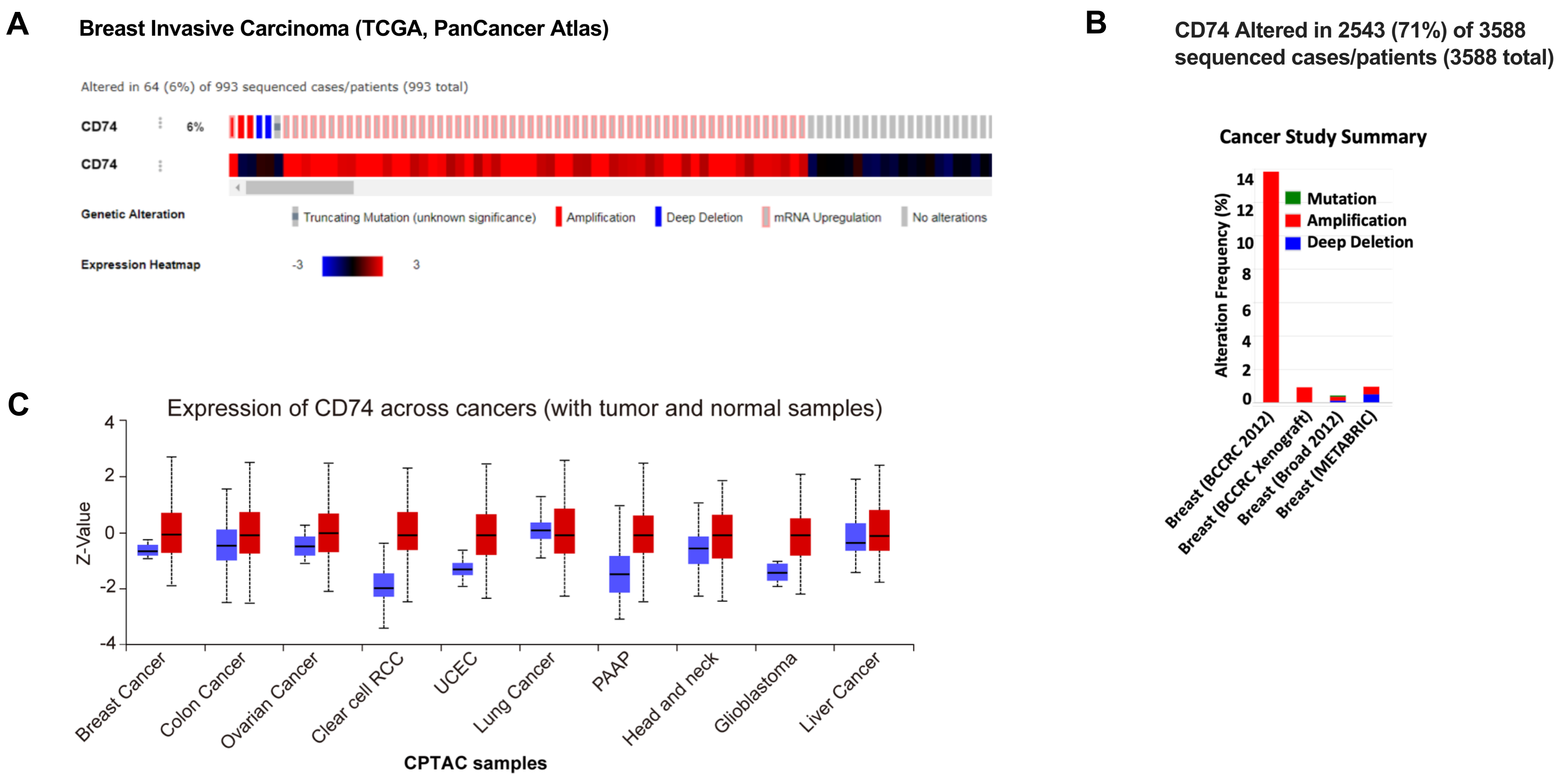


**Supplementary Figure S3**

**A**

```
hCD74  LYQQQGRLDKLTVTSQNLQLENLRMKLPKPPKPVSKMRMATPLLMQALPMGALPQGPMQNATKYGN
mCD74  LYQQQGRLDKLTITSQNLQLESLRMKLPKSAKPVSQMRMATPLLMRPMSMDNMLLGPVKNVTKYGN
rCD74  LYQQQGRLDKLTVTSQNLQLENLRMKLPKSAKPVSPMRMATPLLMRPLSMDNMLQAPVKNVTKYGN
       ************:********.*******..****:*********:.:.*. :  .*::*.*****
```

**B**

**51-** TGFSILVTLL LAGQATTAYF **LYQQQGRLDK LTVTSQNLQL ENLRMKLPKP PKPVSKMRMA TPLLMQALPM -120**

**Peptide 4** QNLQL ENLRMKLPKP PKPVS

**Peptide 3** ENLRMKLPKP PKPVSKMRMA

**Peptide 2** LPKP PKPVSKMRMA TPLLMQ

**Peptide 1** PKPVSKMRMA TPLLMQALPM

**C**

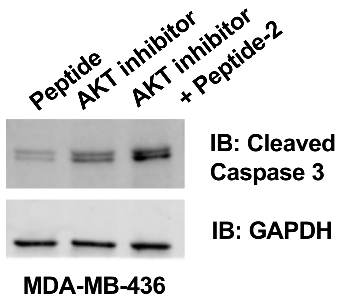


## Supplementary Figure S4
